# HIV counseling and testing practices among clients presenting at a market HIV clinic in Kampala, Uganda: a cross-sectional study

**DOI:** 10.4314/ahs.v17i3.15

**Published:** 2017-09

**Authors:** Joseph KB Matovu, Paul W Bukuluki, David K Mafigiri, Harriet Mudondo

**Affiliations:** 1 School of Public Health, Makerere University College of Health Sciences, Kampala, Uganda; 2 Department of Social Work & Social Administration, School of Social Sciences, Makerere University College of Humanities and Social Sciences, Kampala, Uganda; 3 Market Vendors AIDS Project, Kampala, Uganda

**Keywords:** HCT, practices, market HIV clinic, Uganda

## Abstract

**Background:**

Uptake of HIV counseling and testing (HCT) among informal sector workers is not well documented.

**Objective:**

To assess HCT practices among clients presenting for HIV services at a market HIV clinic in Kampala, Uganda.

**Methods:**

Between August 1 and September 15, 2009, clients presenting for HIV services at a market HIV clinic were invited to participate in the study. Socio-demographic and HCT data were collected from consenting adults aged 16+ years. Descriptive statistics were performed using STATA version 14.1.

**Results:**

Of 224 individuals who consented to the interview, n=139 62 % were market vendors while n=85 38 % were engaged in other market-related activities. Majority of the respondents, n=165, 73.7 %, had ever tested for HIV; of these, n=148,89.7 % had ever tested for 2+ times. The main reasons for repeat testing were the need to confirm previous HIV test results, n=126, 85.1% and the belief that the previous HIV test results were false, n=35, 23.6 %. Uptake of couples' HCT was low, n=63, 38.2 %, despite the fact that n=200, 89 % had ever heard of couples' HCT.

**Conclusion:**

These findings indicate high rates of repeat testing but low rates of couples' HCT uptake in this population.

## Introduction

HIV counseling and testing (HCT) has been promoted on the premise that it provides an entry point into HIV prevention, treatment and care services. Expanding the availability and use of HCT services is a critical step towards the attainment of the UNAIDS global targets dubbed 90-90-90; i.e. 90% of people living with HIV are diagnosed; 90% of those who have been diagnosed with HIV are linked to HIV care; and 90% of those in HIV care have achieved viral suppression^1^. HCT presents an opportunity to share information with clients and promote measures to reduce the risk of HIV infection and transmission^2^. In 1999, Weinhardt et al.^3^ concluded that HCT can reduce sexual risk-taking behaviors among HIV-positive individuals and HIV-discordant couples. In 2003, Allen et al.^4^ found that condom use among HIV discordant couples in Zambia increased from <5% before HCT to over 80% following HCT. Eleven years later, in 2014, a community-based study conducted in 34 communities in Africa and 14 in Thailand found that HCT can reduce HIV incidence by up to 14%^5^. Collectively, these studies demonstrate that HCT has got numerous risk-reduction benefits.

Despite these benefits, up to 40% of people living with HIV are not aware of their HIV status, with the highest proportion reported in sub-Saharan Africa^6^,^7^. A recent UNAIDS report suggests that up to 5.3 million people living with HIV in Eastern and Southern Africa are not aware of their HIV sero-positive status^8^, suggesting a need for interventions to improve HIV diagnosis in this region of the world. Recent guidelines from WHO suggest a need to target couples and male partners for HIV testing as one of the priority populations^9^, highlighting a need for intensified promotion of couples' HIV counseling and testing (couples' HCT.) Couples' HCT provides an opportunity for mutual awareness of HIV status - an important ingredient for couple-based HIV prevention programming - and offers multiple benefits related to timely linkage of couples to appropriate HIV prevention, treatment and care practices^2^. However, uptake of couples' HCT in the general population remains generally low^2^,^9^–^11^. The proportion of those who are aware of their own or each other's HIV status becomes even much lower among those working in the informal sector, including market vendors and other people engaged in market-related activities. This is because such populations are usually considered as highly mobile and hard-to-reach, and hence less targeted by conventional HIV counseling and testing promotion programs^12^. Indeed, a recent study among female market vendors in Kenya found that 11.5% had migrated, changed residence, over county or national boundary in the past year and 39.3% in the past five years. Over one-third 38.3% spent nights away from their main residence in the past month, with 11.4% spending more than a week away^13^. At the time of the study, there were virtually no data available on HCT practices of people working in the market sub-sector, including market vendors and other people engaged in other market-related activities in Uganda.

The objective of this study was to assess HCT practices among clients presenting for HIV services at a market HIV clinic operated by the Market Vendors AIDS Project (MAVAP) in Kampala, the Capital city of Uganda. This study was not intended to assess the effect of MAVAP activities on HCT uptake but to assess and characterize the HCT practices among people accessing HIV services at the MAVAP clinic in order to inform future HCT interventions.

## Methods

### Study design

This was a cross-sectional study conducted among 224 individuals aged 16 years or older, presenting for HCT services at St Balikuddembe market HIV clinic in Kampala, Uganda, between August 1 and September 15, 2009. The study was implemented as part of a large program evaluation of the MAVAP project activities.

### Setting

The Market Vendors AIDS Project (MAVAP) is one of the projects implemented under the umbrella of Development Initiatives International; a registered not-for-profit non-government organization in Kampala, Uganda. The project, which started in 2004, operates in fifteen markets; fourteen in Kampala and one in Mukono district. MAVAP operates an HIV clinic that is located within St Balikuddembe market one of the fourteen markets in Kampala, Uganda. The MAVAP HIV clinic offers HIV/AIDS education, sexually transmitted infections testing and treatment, HCT services, treatment of opportunistic infections, and referral for anti-retroviral therapy among HIV-positive clients, among other services. MAVAP also conducts outreaches to several other market work places on designated “MAVAP days” where services similar to those provided at the HIV clinic are provided to interested individuals. MAVAP serves an estimated market population of 112,700 market operators and 300,000 customers who visit the markets daily. The project receives technical support from an advisory committee that comprises representatives from the Ministry of Health, Kampala Capital City Authority, The AIDS Support Organization (TASO), Uganda Cares and the United Market Vendors Association.

### Study population

The study population was composed of individuals who accessed HIV services at the MAVAP clinic between August 1 and September 15, 2009. Individuals were included in the study if they were 16 years or older and were either involved in market vending or any other market-related activities within St. Balikuddembe market. Prior engagement in a MAVAP-related activity was not a requirement for enrolment into the study. Individuals who did not meet these criteria were excluded from the study.

### Sample size determination

Using the Kish-Leslie formula, with a 5% level of precision, a standard critical value of 1.96 representing 95 % confidence and assuming that 40% of those accessing HIV services at the market-based HIV clinic had ever tested for HIV, we obtained a sample size of 246. After accounting for an estimated 20% non-response^14^, a sample of 307 respondents was obtained.

### Data collection methods and procedures

A trained research assistant introduced the purpose of the study to the clients receiving HIV services at the market HIV clinic, explained the eligibility criteria and allowed individuals to ask questions pertaining to their participation in the study prior to getting enrolled. Thereafter, individuals were asked if they were willing to participate in the study, and those that responded in the affirmative were asked to provide verbal informed consent prior to participation. Individuals that were not interested in the study were informed that this would not affect their access to HIV services offered at the HIV clinic. After obtaining consent, individuals were taken through a screening process to identify those that were eligible for the study. Eligible respondents were administered structured, pilot-tested, interviewer-administered questionnaires that were conducted in Luganda, the main local language that is spoken in the study area. Data were collected on socio-demographic age, sex, education, marital status, etc. and behavioral characteristics, including HIV risk perception and prior HIV testing either individually or together with their partners. The data collection process took an average 40 minutes. It is important to note that respondents were approached at their own convenience, either before or after they had received the HIV services that they came for; so, the implementation of the study at the HIV clinic did not alter service provision in any way. After data collection, all the completed questionnaires were edited in the field to allow for clarification on unclear questions or responses with the respondent before the teams left the field. Edited questionnaires were entered into an EpiData version 3.1 dataset in preparation for analysis.

### Measures

We used the term “HCT practices” to denote the practice of individuals receiving pre-test counseling, HIV test results and associated HIV post-test counseling support. Individuals were asked if they had ever received HCT, and those that responded in the affirmative were asked about how many times they had ever tested for HIV coded as 1, 2–4 or 5+ times and whether or not they had ever tested together with their partners. Prior HCT uptake was assessed by socio-demographic and behavioral characteristics to describe prior HCT practices among clients accessing HIV services at the market HIV clinic. Age was categorized into three categories 16–24, 25–34, and 35+; education was categorized as: primary, secondary and post-secondary education; while respondents were categorized as either being market vendors or engaged in other market-related activities, e.g. customer/buyer. Marital status was categorized as either ‘never married’, ‘currently married’, or ‘previously married’ divorced/widowed/separated. HIV risk perception was assessed using three parameters: Belief that one is personally at risk of HIV infection; belief that one's sexual partner is at risk of HIV infection and belief that market operators are a high-risk group. These parameters were assessed dichotomously, with those holding each belief coded with ‘1’ for “Yes” while those opposing the beliefs coded with ‘2’ for “No”.

### Data analysis

We conducted descriptive analyses i.e. frequencies and percentages to compute respondents' socio-demographic characteristics stratified by sex, HIV risk perception and respondents' prior individual and couples' HIV counseling and testing practices. Due to small numbers, we were not able to compute any inferential statistics beyond the descriptive analyses reported in this paper. Data analysis was conducted using STATA statistical software, version 14.1.

### Ethical considerations

The study was implemented as part of a large program evaluation that was commissioned by the MAVAP project; no ethical review was requested. However, to elicit consent to participate in the study, potential respondents were provided with detailed information about the study and invited to participate. Interested respondents gave verbal informed consent prior to participating in the study. No compensation was provided for participation.

## Results

### Respondents' characteristics

Overall, 238, 77.5% individuals accepted to be interviewed. Of those who were not interviewed, n=69, 80% indicated that they did not have time to participate in the interview or promised to return on another day for interview but did not return while 20% out-rightly refused to participate in the study. This paper is based on 224 individuals for whom complete interview data were available for analysis (Table 1).

**Table 1:** Socio-demographic characteristics of the respondents

Characteristic	Male n=112, %	Female n=112, %	Total N=224, %
**Age-group years**			
16–24	15 13.4	24 21.4	39 17.4
25–34	62 55.4	54 48.2	116 51.8
35+	35 31.2	34 30.4	69 30.8
**Marital Status**			
Never married	50 44.6	36 32.1	86 38.4
Currently married	42 37.5	27 24.1	69 30.8
Previously married	20 17.9	49 43.8	69 30.8
**Education level**			
Primary level	54 48.2	47 42.0	101 45.1
Secondary level	49 43.8	55 49.1	104 46.4
Post-secondary level	09 8.0	10 8.9	19 8.5
**Religion**			
Catholic	42 37.5	28 25.0	70 31.3
Protestant/Church of Uganda	28 25.0	31 27.7	59 26.3
Muslim	30 26.8	29 25.9	59 26.3
Saved/Pentecostal/Seventh Day Adventist SDA	12 10.7	24 21.4	36 16.1
**Occupation**			
Market vendor	72 64.3	67 59.8	139 62.0
Other work	40 35.7	45 40.2	85 38.0

Of these, 139, 62% were market vendors while 85, 38% were engaged in other activities in the market. There were slightly more males (44.6%) engaged in market vending than females (32.1%), although this difference was not significant P=0.49. Majority of those engaged in market vending, n=76, 54.7% had been in this business for 3 or more years data not shown. Majority of the respondents were aged 25–34 years, n=116, 51.8% followed by those aged 35+, n=69, 30.8% and had either primary n=101, 45.1% or secondary education n=104, 46.4%. There were slightly more females with secondary education 49.1% than males 43.8% but this difference was not statistically significant P=0.42.

### HIV counseling and testing practices

Table 2 shows the proportions of respondents that had ever tested for HIV by background characteristics. Nearly three-quarters of respondents had ever tested and received their HIV test results, n=165, 73.7%. Prior HIV testing was reported more by older individuals, e.g. 25–34 years: n=88, 75.9% vs. 16–24 years: n=24, 61.5%; females n=87, 77.7%; currently married respondents n=59, 85.5%; Catholics n=56, 80% and market vendors n=109, 78.4%. Also, prior HIV testing was reported more by those with post-secondary education, n=15, 79% and those who reported that they were market vendors n=109, 78.4% when compared to their counterparts.

**Table 2:** Proportion of respondents who have ever tested for HIV by background characteristics

Socio-demographic characteristics	Total	Number ever tested	Percentage %
**Overall**	**224**	**165**	**73.7**
**Age-group years**			
16–24	39	24	61.5
25–34	116	88	75.9
35+	69	53	76.8
**Sex**			
Male	112	78	69.6
Female	112	87	77.7
**Marital Status**			
Never married	86	56	65.1
Currently married	69	59	85.5
Previously married	69	50	72.5
**Education level**			
Primary level	101	74	73.3
Secondary level	104	76	73.1
Post-secondary level	19	15	79.0
**Religion**			
Catholic	70	56	80.0
Protestant/Church of Uganda	59	38	64.4
Muslim	59	44	74.6
Saved/Pentecostal/SDA	36	27	75.0
**Occupation**			
Market vendor	139	109	78.4
Other	85	56	65.9

Of those who had ever tested, n=148, 89.7% reported that they had ever tested for two or more times. Of these, n=122, 73.9% had ever tested for 2–4 times, while n=26, 15.8% had ever tested for five or more times data not shown. However, there was no significant difference in repeat HIV testing between males and females P=0.98. When respondents were asked why they tested for more than once, n=126, 85.1% reported that they wanted to confirm their previous HIV test results; n=35, 23.6% reported that they thought that their previous HIV test results were false; n=24, 16.2% were encouraged by a friend to repeat the test, while n=9, 6.1% wanted to test for three different times before they stopped testing for HIV. Nearly half of those who had ever tested for HIV n=93, 41.5% reported their most recent HCT site as a MAVAP static clinic or MAVAP outreach session; however, n=89, 39.3% reported taking their most recent HIV test at a government or private facility while n=43, 19.2%, reported that they had their most recent test at other HIV testing facilities within the city.

### Couples' HIV counseling and testing practices

Majority of the respondents, n=200, 89.3% had ever heard of the term “couples' HIV counseling and testing” and nearly all respondents, n=217, 96.9% agreed with the statement, “Testing as a couple is the best option for married people”. However, as shown in Table 3, only a small proportion of those who had ever tested reported that they had ever received their HIV test results together as a couple, n=63, 38.2%. Prior receipt of couples' HCT was much lower among those aged 16–24 years, n=7, 29.2%; females, n=29, 33.3%; those who were divorced/widowed or separated, n=15, 30%; persons with post-secondary education, n=5, 33.3% and Muslims, n=13, 29.5%. Prior receipt of couples' HCT increased with age from 29.2% in those aged 16–24, 38.6% in those aged 25–34 to 41.5% in those aged 35+ years. Prior receipt of couples' HCT was much higher in respondents whose religious affiliation is Saved/Pentecostal or Seventh Day Adventist, n=13, 48.1%; those whose religious affiliation is Protestant/Church of Uganda, n=17, 44.7%; currently married respondents, n=26, 44.1% and men, n=34, 43.6%. There was no significant difference in prior receipt of couples' HCT between those involved in market vending and those engaged in other market-related activities market vending: n=42, 38.5% vs. other activities: n=21, 37.5%, P=0.90.

**Table 3:** Proportion of respondents that have ever received couples' HIV counseling and testing services among ever-tested individuals

Background characteristics	N	No. ever received couples' HCT	Percentage %
**Overall**	**165**	**63**	**38.2**
**Age-group years**			
16–24	24	7	29.2
25–34	88	34	38.6
35+	53	22	41.5
**Sex**			
Male	78	34	43.6
Female	87	29	33.3
**Marital Status**			
Never married	56	22	39.3
Currently married	59	26	44.1
Previously married	50	15	30.0
**Education level**			
Primary level	74	29	39.2
Secondary level	76	29	38.2
Post-secondary level	15	5	33.3
**Religion**			
Catholic	56	20	35.7
Protestant/Church of Uganda	38	17	44.7
Muslim	44	13	29.5
Saved/Pentecostal/SDA	27	13	48.1
**Occupation**			
Market vendor	109	42	38.5
Other	56	21	37.5

When those who had ever received couples' HCT were asked for what motivated them to receive couples' HCT, the majority, n=35, 52.2%, reported that they had always wanted to test together as a couple, n=16, 23.9% reported that this was due to the fact that HCT services were provided in the home or at the work place, n=13, 19.4% reported being motivated by a peer educator, n=11, 16.4% reported that they received an invitation to test as a couple, n=10, 14.9 % reported that both partners attended the same outreach session where couples' HIV testing services were provided, while n=10, 14.9% reported that it was because their partner attended a session where couples' HIV counseling and testing was emphasized.

Of those who have never received couples' HCT services n=37, 23.6 % reported that their partner refused to go with them for couples' HIV testing, n=31, 19.8% had never discussed HIV testing issues with partner, n=17, 10.8% reported that their partners do not see any need for couples' HCT, n=13, 8.3% reported that they just don't want to test with partner, n=10, 6.4% suspect partner to be infected with HIV while n=5, 3.2% fear that their partner might find out that they are HIV-positive. The remaining n=63, 40.1% gave other reasons for not testing with their partners.

## Discussion

In this study of HCT practices among clients accessing HIV services at a market HIV clinic in Kampala, Uganda, we found that nearly three-quarters had ever tested for HIV; of these, nine out of every ten had ever tested for more than once. However, although prior HIV testing was high, only 38% of those that had ever tested reported that they had ever tested together with their sexual partners. The high rates of prior HIV testing among clients accessing HIV services at the market HIV clinic are likely to be a result of the proximity of HIV testing services within the market, since the MAVAP HIV clinic is located within St Balikuddembe market itself. Indeed, 42% of those who had ever tested indicated that they took their most recent test at a MAVAP HIV clinic or MAVAP outreach session. It is also likely that the high rates of repeat testing were a result of prior exposure to MAVAP health education activities or due to exposure to other HCT promotional campaigns at the time. Nevertheless, the presence of the HIV clinic within the market could have been a strong motivator for people to go for repeat HIV testing since they didn't need to incur any travel costs to seek HCT services. However, since 58% of those reporting a previous HIV test sought HCT services from outside the MAVAP HIV clinic; it is likely that convenience alone might not be the sole reason for repeat testing.

Repeat testers usually advance a number of reasons to explain their repeat testing behaviors including preparation for a new relationship^15^; confirmation of previous HIV status, or fear that they might have acquired HIV in the period after the last test^16^. We found that majority of those who returned for repeat testing wanted to confirm their previous HIV test results but nearly a quarter of the respondents returned following concerns that their previous HIV test results were false. These findings suggest a need to emphasize the need for enhanced pre- and post-test counseling messages to individuals seeking HIV counseling and testing at MAVAP clinics to address their concerns on the authenticity of HIV test results received.

Our finding of high rates of repeat testing is consistent with previous studies that have found high rates of repeat testing in high risk groups^15^,^17^,^18^. Indeed, some studies show that it is because of high risk behaviors that individuals seek repeat testing 16 while others show that repeat testing itself is likely to prompt people intohigh risk-taking behaviors^18^,^19^. Compared with first-time testers, MacKellar^18^ found that repeat testers were more likely to report recent risk behaviors and to acquire HIV 7% versus 4%; over 75% of repeat testers who sero-converted acquired HIV within 1 year of their last test. These findings suggest that repeat testers are more likely to engage in higher risk behaviors than first-time or non-testers. However, since we did not collect sexual risk behavior data, there is need for further research to examine the sexual risk-taking patterns of market operators in order to fully characterize the repeat testing behaviors observed in this population. This will help to inform the development of appropriate HIV prevention interventions targeting this group.

The finding that couples' HCT uptake was low in this population is consistent with findings from other studies regarding couples' HCT in general^21^–^23^, indicating limitations in improving joint awareness of couple HIV status through couples' HCT. Several reasons have been reported to inhibit couples' HCT uptake, including low risk perception among couples coupled with the belief that a partner's HIV test results should be similar to a spouse's; also known as ‘HIV testing by proxy’^24^, fears of receiving HIV-discordant results, low levels of male involvement and fears of marital dissolution resulting from joint awareness of HIV test results especially in the event that one or both partners are HIV-positive^25^. Since there are high rates of re-testing in this population, it is important that all tested individuals are encouraged to share their HIV test results with their partners; where appropriate, with assistance from a professional counselor. Otherwise, any interventions aimed at improving couples' HCT uptake in this population should address the apparent fears expressed by the participants but also explore alternative ways of encouraging communication between partners about couples' HIV testing^26^ in the hope that this discussion will arouse interest in the partner to accept couples' HCT.

The findings of this study should be interpreted with caution. In the first place, this study was conducted in a market setting with very close proximity to HIV testing services. It is likely that the uptake rates reported in this setting, including the high levels of re-testing, might be due to the proximity of services rather than a dire need to repeat the test. However, since nearly 60% of those who tested for HIV sought the services elsewhere, it is likely that our findings might reflect genuine interests for re-testing in this population. Secondly, the findings reported in this paper are based on individual self-reports that are subject to reporting bias. It is also likely that our study could have targeted a highly self-selected group of repeat testers; and if this were true, then the repeat HIV testing rates reported in this paper might not be representative of the HIV testing behaviors of market vendors or other people engaged in market-related activities. Because there are no prior studies on this subject, we don't know the extent to which our study population differs from or is similar to other informal sector workers; or whether those interviewed were similar to or different from the average market vendors or other people engaged in market-related activities in Uganda. Further research is warranted in other markets to obtain data necessary to fully characterize the HIV testing behaviors of these informal sector workers.

Given the relative lack of HIV data on the study population, this study would have benefitted from collecting data on the risk profiles of clients accessing HIV services at the market HIV clinic, including their sexual risk behaviors, biomarkers of HIV risk exposure e.g. history of sexually transmitted infections, and other factors that increase their vulnerability to the risk of HIV infection. A recent study among female market vendors in Kenya found that 25.6% were HIV-positive13; suggesting that people engaged in market vending and other market-related activities could be at a heightened risk of HIV infection. Future research should include a component that explores the sexual risk-taking characteristics of all people involved in the informal sector in order to inform the design of target-specific interventions. It is also important to note while the proportion of those who had ever received couples' HCT was expressed out of those that had ever tested for HIV, we do not know if all them were involved in any sexual relationships or living with any sexual partners at the time they were tested for HIV. This is because we did not include any questions asking respondents whether or not they were living with any sexual partners at the time they tested for HIV. For this reason, the reported proportion of prior couples' HCT uptake should be interpreted with caution. Nevertheless, despite these limitations, our study is unique in that it is the first study to document HIV testing practices of individuals accessing HIV services at a market HIV clinic, and provides useful information to guide HIV prevention planning for this rather neglected but important HIV risk group.

## Conclusion

Our findings show high rates of prior HIV testing, high rates of re-testing but low rates of couples' HCT uptake in this population, suggesting that interventions that enhance increased uptake of couples' HCT services among informal sector workers are urgently needed.
